# Triple mediation of attitude to bridge transformational leadership on organizational citizenship behavior

**DOI:** 10.1371/journal.pone.0281220

**Published:** 2023-02-02

**Authors:** Heni Yuwono, Anis Eliyana, Agung Dharmawan Buchdadi, Hamidah, Nurul Iman Abdul Jalil

**Affiliations:** 1 Universitas Negeri Jakarta, East Jakarta, DKI Jakarta, Indonesia; 2 Department of Management, Universitas Airlangga, Surabaya, East Java, Indonesia; 3 Department of Psychology and Counseling, Universiti Tunku Abdul Rahman, Kampar, Perak Darul Ridzuan, Malaysia; Al-Ahliyya Amman University, JORDAN

## Abstract

This study was conducted to determine the Organizational Citizenship Behavior (OCB) of correctional officers at the Super Maximum-Security Prison in Indonesia which is influenced by Transformational Leadership (TL) either directly or indirectly through Affective Organizational Commitment (AOC), Job Satisfaction (JS) and Job Self-Efficacy (JSE). This research was conducted on 224 prison officers as a sample size. Data was collected by means of a questionnaire distributed via Google Form. Robustness was built in two stages; the first stage was through a try out of research instruments and the second stage was through data collection which was done with the time lagged method. Furthermore, the data were analyzed using Structural Equation Modeling (SEM) with the help of AMOS 24 software. The results of this study indicate that triple mediation consisting of AOC, JS and JSE fully mediates the effect of TL on OCB. The novelty of this research lies in the role of triple mediation as the focuses of the study. Furthermore, the triple mediation has been proven to fully mediate the effect of TL on OCB thus may serve as empirical evidence that contribute to theoretical and practical developments in the fields of Human Resource Management and Organizational Behavior.

## 1. Introduction

The Correctional System is an order containing direction and limits on how to foster Correctional inmates in improving themselves, realizing mistakes, and not repeating criminal acts. It is hoped that they can later be accepted by the community, play an active role in society, and live naturally as good citizens and be responsible for the state. In general, the function of the Correctional Institution is as a place to conduct coaching to improve the quality of prisoners. High level convicts with cases including international crimes, terrorism, narcotics trafficking syndicates, and serious criminal crimes are held at the Super Maximum-Security Prison, Nusakambangan. Here, a criminal confinement program and training for high-risk prisoners is carried out to encourage stability and security. According to the data obtained, the numbers of officers at the Nusakambangan prison complex are limited and their job demands are high, so an extra role is needed among the officers to meet the standards of the Super Maximum-Security Prison.

The problems that occur in prisons are not as simple as imagined, especially regarding overcapacity which cannot be overcome simply by adding or building new prisons. This has an impact on social problems, such as disruption of order, security, conduciveness, and the functionality of prisons. Overall, the prison in Nusakambangan has a capacity of 2027 inmates, but is inhabited by 2365 inmates with criminal cases of terrorism, narcotics and general crimes [1]. In other words, there is an overcapacity of 17%.

This is worsened with the unbalanced ratio of officers to prisoners being fostered. Comparison of the number of prisoners and officers (security units) that are not proportional to the number of prison residents. The number of officers is 395 people, with details of 224 officers in the technical field and 171 administrative officers based on Employment Information System. From these data, it can be seen that the ratio between prisoners and prison officers is 10.5 to 1. Ideally, a prison with supervision like the prison in Nusakambangan, 1 officer supervises 3 inmates. According to the Bureau of Justice Statistics 2020, the ratio between inmates and prison officers is 3 to 1 [2]. When compared, the ratio of prison officers and inmates in prisons in Nusakambangan is not ideal. The small number of officers at the prison in Nusakambangan certainly affects the implementation of duties and functions in carrying out guidance and security.

This condition resulted on the officers’ average minimum working hours of 32.5 hours per week with three shifts that alternate over a 24-hour period. Due to long working hours, heavy workloads, and lack of experienced human resources, officers often have to carry out additional work. For example, officers can function as guardians and security officers but also serve as a team for the Emergency Response Team. With so many work activities that need to be completed, there must be factors that encourage employees to perform these tasks well. A person’s behavior is indeed outside the responsibility of the organization and the laws and regulations, but if it is carried out by employees within the scope of an organization, it can improve the function of the organization which is also known as OCB. OCB is defined as “an individual behavior that is discretionary, not directly or explicitly recognized by the formal reward system and in aggregate promotes the effective functioning of the organization” [3]. That way, OCB is expected to encourage progress and achievement of company goals. From several previous studies, it can be concluded that OCB is a behavior that is expected to be possessed by employees, this behavior is considered beneficial to the organization and cannot be added to formal obligations or as a recommendation. If we look further, OCB is a factor that contributes to the overall work of the organization. This is no exception in the Nusakambangan prison complex.

In this regard, the researcher focuses on OCB, which is a multidimensional concept that includes all aspects of voluntary behavior, namely exceeding job requirements through the influence on leadership in an organization [4]. Leadership has been shown to be able to influence individual behavior in the workplace, and effective leaders bring more benefits to the workplace and are able to have the ability to motivate employees to perform behaviors that produce positive outcomes in the workplace. TL is considered to be the most effective leadership approach and is considered to have a positive impact not only on organizational development, but also on relationships with followers and the development of individual followers. TL is also known to be more open-minded and visionary, which can be a motivation for employees to work beyond expectations [5]. Officers with TL tend to have higher performance and subsequently affect the level of OCB of these officers. This is because they have higher motivation than officers with other leadership styles.

The TL refer to the leader’s efforts to motivate his officers to work with high performance, resulting in a professional organizational culture and increasing welfare levels that can encourage the affective work of officers in encouraging organizational commitment [6]. Organizational commitment will refer to an individual’s psychological attachment to the organization, and showed that of the three components of organizational commitment (affective, normative, and calculative) [7]. Furthermore AOC has more desirable outcomes for the organization [8]. AOC will lead to an employee’s emotional attachment which is identified with involvement in the organization [9]. in addition, AOC can occur to employees when they want to be part of the company based on the emotional bond that the employee has [10]. In addition, TL will also enable individuals to achieve organizational goals, be more enthusiastic at work, and have a sense of belonging to the organization which can affect affective commitment and furthermore they are more likely to be involved in it with extra role behavior or OCB to pay the organization [11].

In addition, JS is also found to have a correlation with citizenship behavior. TL behavior can affect JS through employees’ perceptions of their transformational leaders, because these leaders will increase employee expectations and recognition of their work and increase employee JS through TL behaviors such as individual attention, intellectual stimulation, and motivation [12]. Employees who are satisfied with their work are ready to do extra mile work for the purpose of their organization and to help their colleagues. The existence of positive feelings also increases their desire to interact with other officers and is happy to help so that organizational citizenship attitudes emerge. Employees will show their emotions, whether they are satisfied with the job or dissatisfied, and it is known that they measure their level of satisfaction through various factors such as wages, welfare, proper controls, salary increases, job descriptions and coworkers [13].

In addition, it is known that JSE can be a variable acting as a resource at the time of organizational change, which in turn increases changes in attitudes and supportive behavior [14]. This can be influenced by the resources that come from TL that can build a strong reserve of resources. When employees receive resources from their superiors during organizational change, they will be better prepared to deal with the demands of the situation and more supportive [14]. Self-efficacy is one of the valuable resources, especially in the context of change, individuals who develop self-efficacy believe that they are able to handle uncertainty and change that also leads to positive behaviors such as OCB. Individuals with high JSE will dare to take risks and maximize their ability to work, including leading to a willingness to do work that is beyond their obligations such as OCB [15].

As can be seen from the description above, OCB is usually very important for the entire organization. In this study, we will examine the importance of OCB in government agencies, namely the Nusakambangan Super Maximum-Security Prison. The importance of OCB in the Super Maximum-Security Prison lies in the lack of human resources and the workload and high work risk; therefore, OCB is needed so that employees can achieve organizational goals together. Research with the topic of leadership and looking for the influence of leadership aspects TL on OCB is rarely studied in correctional institutions. In addition, the research model that is carried out proposes mediation that comes from organizational commitment, JS, and JSE.

This study modifies the existing model by using AOC, JS and JSE as mediation. This is a novelty in this research because it is rare to find research on OCB that focuses on the role of TL with AOC, JS and JSE as mediation, especially in the context of overcapacity prisons with super maximum security. The influence of a transformational leadership style and the presence of high commitment, positive job satisfaction, and appropriate self-efficacy will lead to extra-role behavior.

Based on the description of previous research and phenomena that occur in the field, in capturing the phenomena that occur in Correctional Institutions, researchers are interested in conducting research on Super Maximum Security Prison officers regarding the existence of OCB in officers who are influenced by TL in the organization with several variable that becomes the intermediate variable is AOC, JS and JSE.

## 2. Literature review

### 2.1 Theoretical foundation

#### 2.1.1 Transformational leadership

TL is one leadership style that is effective in encouraging positive behavior to be able to create significant changes for followers and also the organization, which also has the ability to be able to direct changes in strategy, structure, mission, and organizational culture to promote product and work innovation [16]. TL is considered to be the most effective leadership approach and is considered to have a positive impact not only on organizational development, but also on relationships with followers and the development of individual followers. That is because TL also refers to leaders who encourage their subordinates to go beyond their own self-interest for the good of their organization [17]. TL is also known to be more open-minded and visionary, which can be a motivation for employees to work beyond expectations [5]. Thus, TL is simply stated as a way of influencing others that allows them to experience change and growth, and prepare themselves to become the next leader through ready change and a work environment full of innovation [18].

#### 2.1.2 Affective organization commitment

The concept of organizational commitment which was originally pioneered by Mowday by dividing organizational commitment into three forms, namely continuous commitment, normative commitment and affective commitment [19]. Among the three conceptualizations, AOC is known to have the strongest overlap with the definition of operational attitude which is also stated to be one of the components most closely related to well-being in the workplace [6]. Affective commitment will lead to an employee’s emotional attachment which is identified with involvement in the organization [9] and affective commitment can occur to employees when they want to be part of the company based on the emotional bond that the employee has [10]. Through AOC, organization can gain the ability of superior human resources that can protect their existence in the organization [20], by maintaining the existence of superior human resource capabilities in companies such as companies can have resilience in maintaining / creating longer sustainable competitive advantage.

#### 2.1.3 Job satisfaction

JS is one of the elements that influence organizational results [21]. JS is defined as an emotional reaction and behavioral expression towards work resulting from individual assessments of work performance, office environment, and work life [22]. JS can be briefly defined as a combination of positive or negative feelings that employees have about their jobs, and in Locke’s most frequently cited definition describes it as "a pleasurable or positive emotional state resulting from an appraisal of one’s job or work experience" [23]. JS is also expressed as a form of optimistic emotional state arising from one’s work, which is related to how a person likes his job, an employee’s view of his work positively, and JS is an observable expression of affective reactions to certain jobs [24].

#### 2.1.4 Job self-efficacy

Theory of JSE which is known that individuals with JSE will be able to influence the goals [25] that individuals choose for themselves; the level of commitment shown by workers to work; and attitudes toward learning and handling complex tasks [26]. In line with the COR theory, self-efficacy will act as a resource in times of organizational change, which in turn increases changes in attitudes and supportive behavior [14]. Self-efficacy is defined as the belief that a person will be able to perform in a certain way to achieve certain goals, in other words, individuals with high levels of self-efficacy will be involved in completing their tasks while individuals with low self-efficacy will do the opposite or will only be a failure for the organization [5]. Self-efficacy can also be used as a complex cognitive assessment of a person’s or individual’s ability to choose the behavior or actions needed to achieve certain goals in the future [27]. Thus, JSE will refer to people’s beliefs in their ability to mobilize the cognitive and behavioral resources needed to exercise control over environmental events", or in the context of an organizational environment self-efficacy is expressed as employees’ perceptions of their ability to work well good [28].

#### 2.1.5 Organizational citizenship behavior

Studies investigating OCB show that workers’ willingness to fulfill formal duties and roles is not sufficient to predict organizational effectiveness or is expressed as a voluntary aspect that predicts organizational effectiveness and enables efficient management/leadership [29]. The concept of OCB defined as “an individual behavior that is discretionary, not directly or explicitly recognized by the formal reward system and in aggregate promotes the effective functioning of the organization” [3]. Volunteering to engage in extra behavior is needed because it contributes to maintaining and enhancing the social and psychological context that supports task performance [30]. It will also support employees to achieve high-quality results in the workplace [31]. The definition of OCB is expressed as voluntary behavior and personal choice whose initiatives are not directly related to the organization’s formal reward system but can increase the effectiveness of the organization as a whole [32]. So, it is stated that OCB refers to discretionary behavior that goes beyond formal job descriptions and increases organizational effectiveness which also adds meaning to employees’ daily work by stimulating personal development and contribution to their organization [33].

### 2.2 Hypothesis development

#### 2.2.1 Transformational leadership affects organizational citizenship behaviour

OCB is behavior that employees do outside of routinely recognized duties and job assignments, and this behavior is organizationally desirable because of its relationship with organizational effectiveness. In general, leaders have a strong effect on followers’ organizational behavior including OCB which is no exception. This study examines the relationship between leadership and OCB that has considered TL. According to previous study, TL can motivate their followers to do more than work tasks and challenge the status quo, because they can expand their efforts related to the daily work of their employees to go beyond the requirements and job descriptions, so it shows OCB [4]. The study assumes that TL will make them see it as more useful, affect the way employees think about their work, work passionately and meaningfully, and it is known that it will affect the extent to which they are involved in OCB [4]. Through TL, followers will be able to motivate followers by communicating the need to prioritize and internalize organizational interests above their individual interests, which causes intrinsic motivation to lead to followers’ willingness to contribute to organizational goals, without expecting direct personal and tangible rewards [29]. Thus, they are willing to do more than expected by the description of formal organizational roles such as OCB. Previous studies maintain that a positive relationship between TL and OCB has been empirically supported [4, 29]. the behavior that characterizes TL will have a positive effect on followers’ OCB [34]. TL characteristics will ’run the conversation’, treat their followers fairly and show that they trust them, along with taking an interest in their individual well-being, are more likely to see similar behaviors replicated by the workforce which will generate enthusiasm among followers to display prosocial activities. such as OCB, because they set an example for them to follow [34].

**H1. Transformational leadership affects organizational citizenship behavior**

#### 2.2.2 Transformational leadership affects affective commitment

TL will find meaning in ordinary activities, which leads to fostering a strong affective commitment from followers to organizational goals [35]. According to the study, one of the biggest characteristics of TL is the ability to motivate their followers and increase their organizational commitment, which in turn leads them to show extraordinary performance. Thus, the efficiency of a leader who adopts TL behavior (e.g., participation and decision making, intellectual stimulation, individual consideration, individual support, symbols and values of professional practice) is known to be the main determinant of affective commitment [6]. In addition, TL has a tremendous influence on followers and their success in building commitment, which shows a transformational leader change and creates meaning for employees who promote AOC [8]. TL will encourage subordinates to do more than expected, emphasize the individual needs and personal development of followers, underline the importance of respecting and appreciating subordinates, as a result followers feel loyalty and respect for TL. In other words, these leaders transform employees by increasing motivation and commitment, and empowering them to achieve organizational goals [8]. So that previous research has revealed TL specifically related to affective commitment [6, 8, 35].

**H2. Transformational leadership affects affective commitment**

#### 2.2.3 Transformational leadership affects job satisfaction

A successful TL style will be able to contribute and increase employee motivation to achieve the mission and goals of the organization [21]. The research has also emphasized that JS is one element that influences organizational outcomes. Therefore, it is stated that TL is the key to improving and developing the overall vision and goals for the organization by increasing JS. Meanwhile, TL will support and motivate their employees to achieve human needs which is also important to promote JS [36]. TL behavior can affect JS through employees’ perceptions of their transformational leaders, because these leaders will increase employee expectations and recognition of their work and increase employee JS through TL behaviors such as individual attention, intellectual stimulation, and motivation [12]. In addition, it is known that the participatory decision-making style practiced by TL can also give employees higher JS. It is also supported by previous studies, which stated that TL has an impact on increasing JS [21, 36, 37].

**H3. Transformational leadership affects job satisfaction**

#### 2.2.4 Transformational leadership affects job self-efficacy

TL can provide JSE as a resource to followers in providing the best job results. Research has indeed shown that self-efficacy is one of the TL mechanisms in increasing supportive attitudes of change by giving followers confidence that change can achieve the desired results [14]. The leader can serve as a role model for employees by demonstrating a self-confident model that helps individuals feel capable of coping with the high demands of change. TL will be able to encourage employees to rethink the demands of change, demands that may seem less threatening and employees may feel better able to handle those challenges, and TL can also provide these employees with available support if they need help to improve their JSE [38]. Self-efficacy can act as a resource needed during organizational change, which in turn increases supportive attitude and behavior changes which will become one of the valuable resources capable of dealing with uncertainty and change [14]. According to this study, TL can affect self-efficacy as a resource to followers through the process of increasing a supportive attitude of change by giving followers confidence that change can achieve the desired results. This idea is supported by previous studies which suggested that TL is an important determinant of self-efficacy in followers [14, 18, 28].

**H4. Transformational leadership affects job self-efficacy**

#### 2.2.5 Affective commitment affects organizational citizenship behaviour

*AOC is known to be determined by the employee’s personal choice to remain committed to the* organization through some emotional identification with the organization that shows a positive attitude towards the organization [39]. Affective commitment will show that the employee’s attitude as an individual is related to the personal values that the person brings to the organization. In terms of the relationship between affective commitment and OCB, many studies have shown that affective commitment has a significant and positive relationship with OCB in various organizational settings [35]. Based on the social exchange framework, it would make sense that emotionally attached individuals can show reciprocal behavior of OCB, meaning that in high-quality social exchange relationships indicated by high levels of affective commitment, employees are more likely to engage in OCB because they feel a relational obligation to produce positive outcomes that benefit their relational partners [35]. Supported by previous research which has stated that affective commitment determines variables that AOC such as OCB [40–42]. This can occur OCB, because through affective commitment will imply multidimensional, including employee loyalty to the organization, their willingness to make efforts on behalf of the organization, the level of goals and conformity of values with the organization, and their desire to maintain membership [42].

**H5. Affective commitment affects organizational citizenship behavior**

#### 2.2.6 Job satisfaction affects organizational citizenship behaviour

JS is expressed as an emotional reaction and behavioral expression towards work resulting from an individual’s assessment of the office environment, work performance, and work life, and as a pleasant emotional state resulting from the assessment of one’s work as an achievement or facilitation of achieving one’s job value [22]. Furthermore, individuals with higher levels of JS will show more pro-social behavior, such as OCB. OCB is known to provide dimensions that are considered quite passive or reactive in their orientation, such as compliance with organizational procedures [43]. Thus, individuals with higher JS will lead to OCB which refers to individual behavior that is influenced by an interpersonal trust that is able to facilitate organizational operations by performing tasks outside the organizational prerequisites, which are not directly recognized by the formal reward system. Employees who enjoy a higher level of JS will be able to show a higher level of OCB [22, 44].

**H6. Job Satisfaction affects organizational citizenship behavior**

#### 2.2.7 Job self-efficacy affects organizational citizenship behaviour

Self-efficacy theory can state that belief will affect their ability to predict an effort that they will then make, and the extent to which they persist in the face of seemingly insurmountable challenges [45]. JSE can influence how they think, how they behave, how people feel, and what motivates them, because self-efficacy is defined as individuals’ persuasion about their own ability to achieve the goals that have been set [46]. It will make individuals with the right JSE led to positive behavior for organizations such as OCB. Those with high self-efficacy tend to be more confident because they believe they can complete their work, and this is related to the belief that they are able to perform the expected actions well [47]. Furthermore, individuals with high JSE will dare to take risks and maximize their ability to work, including leading to a willingness to do work outside of their obligations such as OCB [15]. Previous studies have confirmed that JSE leads to OCB [26, 47, 48].

**H7. Job self-efficacy affects organizational citizenship behavior**

#### 2.2.8 Transformational leadership mediates the effect of affective commitment on organizational citizenship behaviour

The role of TL is very important in describing it as a process of exchange of leaders and followers helping each other to advance to a higher level of morale and motivation [18]. The basic tenets of social exchange theory suggest that individuals will have a tendency to reciprocate dyadic pairs with behaviors that are beneficial to them, and in an organizational context. This behavior can be demonstrated through OCB, where employees carry out activities that are not part of their responsibilities but help the organization to achieve its goals [35]. Furthermore, the role of TL will help employees’ OCB through direct results of the affective commitment they feel towards the organization. TL will also enable individuals to achieve organizational goals, be more enthusiastic at work, and have a sense of belonging to the organization which can affect affective commitment [11]. So, it is stated that when employees have high-quality social exchange relationships from the influence of TL on the organization, their affective commitment increases, and they are more likely to engage in extra role behavior or OCB to pay the organization.

**H8. Transformational leadership mediates the effect of affective commitment on organizational citizenship behavior**

#### 2.2.9 Transformational leadership mediates the effect of job satisfaction on organizational citizenship behaviour

JS is related to TL in terms of employees taking part in organizational decision-making processes [29]. TL is known to be able to create significant changes for followers and organizations through creating the ability to direct changes in strategy, mission, organizational structure and culture, which can promote innovation [27]. Open communication between managers and employees, and employee participation from the influence of TL in the decision-making process is known to increase JS. Employees who work under a TL influence will be more satisfied, engaged, empowered, motivated, trusted and committed to their organization and exhibit less withdrawal behavior [49]. Employees’ attitudes towards the workplace are reflected in increasing JS and trust, which then affect OCB. Thus, increasing TL predicts JS and increases OCB.

**H9. Transformational leadership mediates the effect of job satisfaction on organizational citizenship behavior**

#### 2.2.10 Transformational leadership mediates the effect of job self-efficacy on organizational citizenship behaviour

The relationship between JSE and positive reactions such as OCB will be mediated by TL. This mechanism suggests that TL will be able to increase the JSE of their followers while creating positive behavior, and increase JSE, which in turn, encourages intentions to support OCB. This mediation mechanism is explained by the COR theory [14]. TL will be able to provide self-efficacy as a resource to followers. Previous study has shown that self-efficacy is one of the TL mechanisms that can increase supportive attitude change by giving followers confidence that change can achieve the desired results [14]. Furthermore, it is this positive influence that creates employees to voluntarily perform extra roles such as OCB. Thus, increasing TL predicts JSE and increases OCB.

**H10. Transformational leadership mediates the effect of job self-efficacy on organizational citizenship behavior**

**Fig 1:**
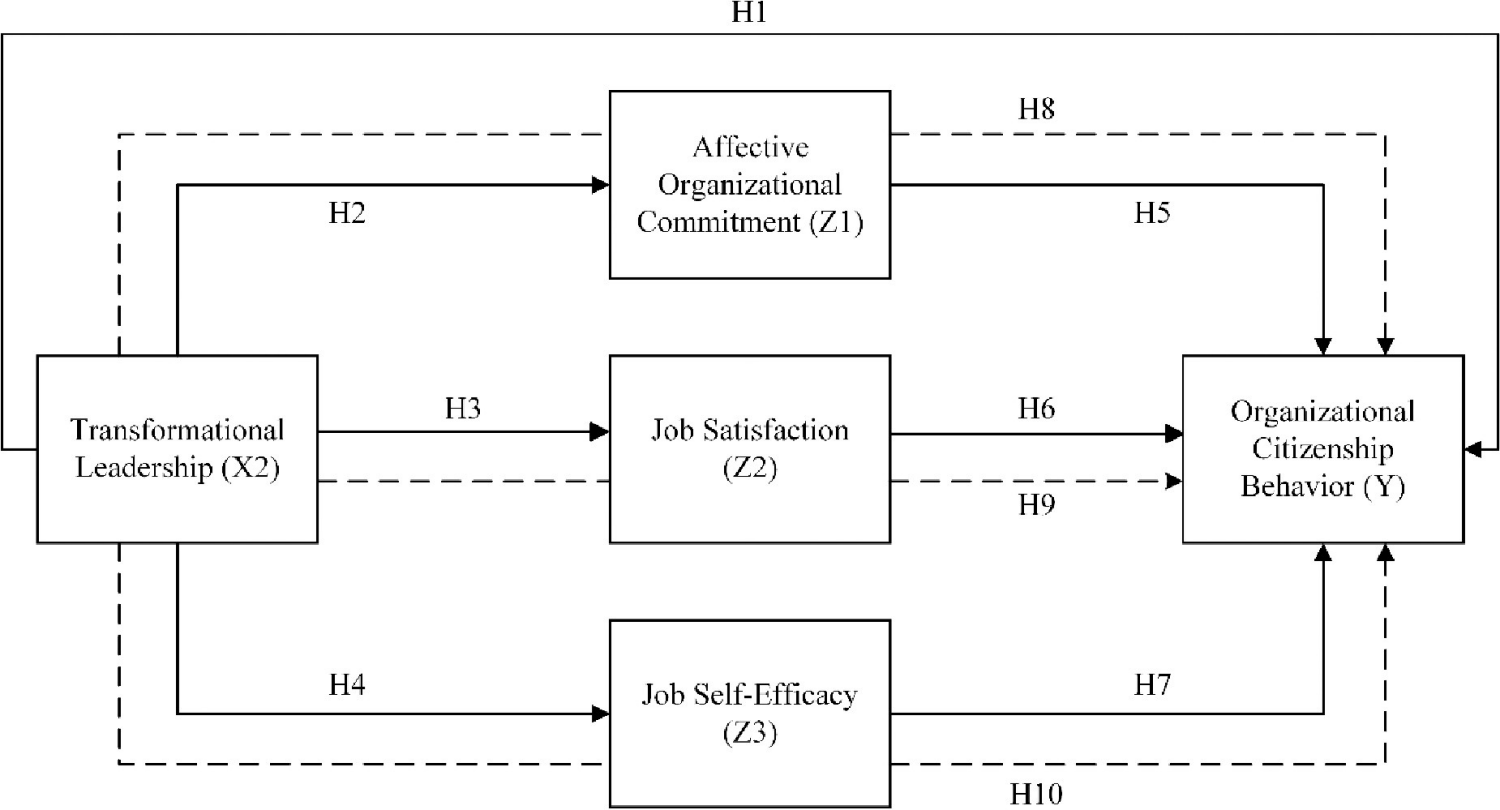
Conceptual framework.

All of the hypotheses are conceptualized in the following framework (Fig 1):

## 3. Research method

### 3.1 Research approach

This research approach uses a quantitative approach, and the design of this study is causal research that aims to test hypotheses about the effect of exogenous variables (TL) on endogenous variables (Organization Citizenship Behavior) through a mediating role (AOC, JS and JSE). at the Nusakambangan Super Maximum-Security Penitentiary.

### 3.2 Robustness of research instruments

In measuring the variables studied, this study used several instruments that were adopted and developed from previous researchers which were measured with a Likert scale of 1–5. The TL instrument consists of 20 indicators [50]; AOC consists of 8 indicators [51]; JS consists of 10 indicators [52]; JSE consists of 10 indicators [53]; and OCB consists of 15 indicators [54].

Furthermore, to ensure that the research instruments truly reflect the actual conditions of the respondents and the research context, a try out was conducted on 50 respondents who were not the sample of this study. The try out was intended to strengthen the robustness of the research instrument with the help of SPSS Statistic 24 software. The results of the try out show that all instrument indicators are valid and reliable.

The validity of the instrument is indicated by the correlation value for each measurement item on all variables > 0.30 and is also significant at the level of significance = 5%. In addition, the reliability of the instrument is indicated by the value of Cronbach’s alpha value > 0.60. Thus, the preparation of the statement items used to measure the variables used is declared valid and can be used for further analysis. In addition, it can also be stated as reliable and trustworthy as a consistent measuring tool.

### 3.3 Robustness of data and sample collection techniques

The sample of this study which were amounted to 224 were determined by certain criteria based on purposive sampling technique. The criteria are that the respondents are permanent officers; guard officers who have the functional task of maintaining order, security, and prison conditions; have worked for more than 2 years; and work in shifts (there are 3 shifts in 1 working day). The data collection was done with time lagged method since it was carried out 3 times in a span of 1 month through Google Form. The first month of data collection was for exogenous variables, namely TL. Next, the second month of data collection was for the interverning variables, namely JS, AOC, and JSE. Finally, data collection for endogenous variable, that is OCB was done on the third month. The time lagged method was intended to strengthen the robustness of the data obtained so that the results of the analysis reflect the respondent’s situation and the research context.

Before completing the questionnaires, respondents were informed that their identities would remain anonymous and that the data would only be used for research and publication purposes. The chief executive officer consented in writing on behalf of all respondents as comply with the organization’s policy. The Research Ethics Committee of Universitas Airlangga (LIPJIPHKI) determined that no ethical approval was necessary to validate a non-interventional study. Once obtained, the data were then analyzed using the Structural Equation Model (SEM) technique using AMOS 24 software. Structural Equation Modeling or SEM is a statistical technique that allows testing of relatively complex constellations of models simultaneously [56].

## 4. Results and discussion

### 4.1 Results

Respondents in this study consisted of 224 prison officers in Nusakambangan. The characteristics of the respondents are shown in Table 1.

**Table 1:** Characteristics of respondents.

Characteristic	Classification	N	Percentage
Gender	Male	206	92%
	Female	18	8%
	Total	224	100%
Age	20–30 years old	128	57%
	31–40 years old	52	23%
	41–50 years old	27	12%
	> 50 years old	17	8%
	Total	224	100%
Tenure	< 1 Years	72	32%
	1–3 Years	46	21%
	4–6 Years	5	2%
	7–10 Years	7	3%
	11–14 Years	48	21%
	> 15 Years	46	21%
	Total	224	100%
Higher Education	Senior High School	140	63%
	Diploma	10	4%
	Bachelor	64	29%
	Master	10	4%
	Total	224	100%

Multicollinearity identification is performed prior to evaluating the Measurement Model and Structural Model. Multicollinearity is the degree to which exogenous variables are interrelated. A relationship that is excessively high between exogenous variables results in effect redundancy, therefore a variable whose influence should be significant may become insignificant [55]. Multicollinearity can be identified using a correlation matrix [56]. A correlation value of 0.80 or above in the correlation matrix implies multicollinearity [55]. The correlation matrix among indicators (sample correlation matrix) produces the lowest value of 0.156 and the greatest value of 0.789, as determined by the multicollinearity detection. Therefore, it may be inferred that there is no multicollinearity in this research model.

**Fig 2:**
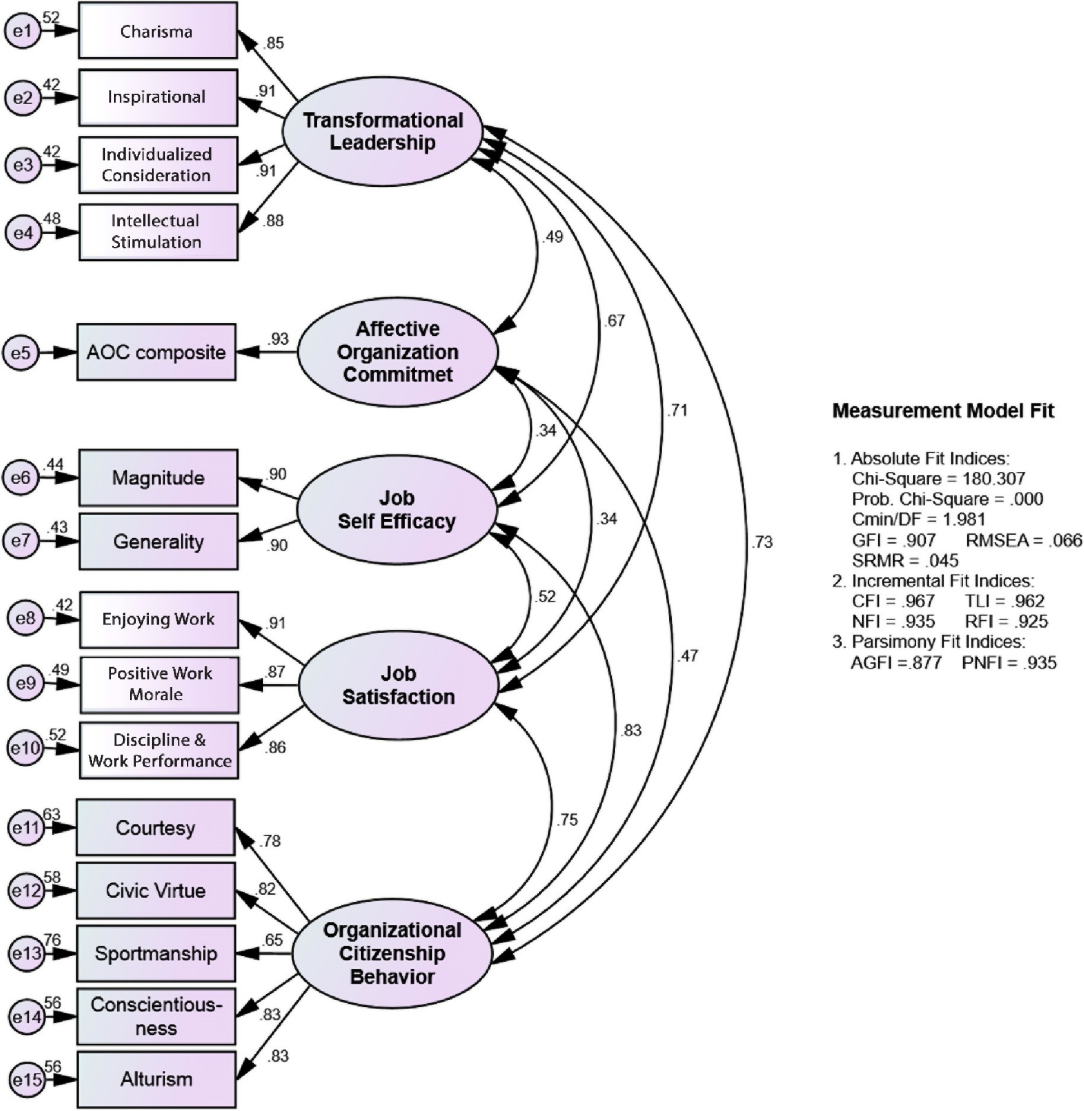
Evaluation of the measurement model.

The summary of the results of the measurement model suitability test is presented in Fig 2.

Table 2 shows the results of the evaluation of the suitability of the measurement model yielding criteria that are all acceptable, namely good fit and marginal fit, except for the probability chi-square, so the measurement model can be accepted. The chi-square probability value in the measurement model produces a value of 0.000, not greater than 0.05. This condition does not mean that it belongs to the category of poor fit.

**Table 2:** Fit measure for the measurement model.

*Fit Measure*		*Critical Value*	*Measurement Model*	
			*Index value*	Decision
*Absolute Fit Indices*	Probability	> 0,05	0,000	*Even good fit*
	Cmin/DF	≤ 2,00	1,981	*Good fit*
	GFI	≥ 0,90	0,907	*Marginal fit*
	RMSEA	≤ 0,08	0,066	*Good fit*
	SRMR	≤ 0,05	0,045	*Good fit*
*Incremental Fit Indices*	CFI	≥ 0,95	0,967	*Good fit*
	TLI	≥ 0,95	0,962	*Good fit*
	NFI	≥ 0,90	0,935	*Good fit*
	RFI	≥ 0,90	0,925	*Good fit*
*Parsimony Fit Indices*	AGFI	≥ 0,90	0,877	*Marginal fit*
	PNFI	≥ 0,90	0,935	*Good fit*

Table 3 shows that in the measurement model, each indicator on the exogenous construct, intervening construct, and endogenous construct consisting of TL variables, affective organization commitment, JS, JSE, and OCB, all have factor values. loading is greater than 0.50 and the AVE value is also greater than 0.50, so that these indicators are valid in forming constructs and can be used to build models, and the variables produce construct reliability values greater than 0.70, respectively. Thus, it can be concluded that these indicators are reliable in reflecting exogenous constructs, intervening constructs, and endogenous constructs.

**Table 3:** Construct validity and construct reliability.

Variable	Indicator	Initial Model		AVE	Construct Reliability	Decision
		Factor Loading	Decision			
*Transformational Leadership*	Charisma	0,855	Valid	0,788	0,937	Reliable
	Inspirational	0,909	Valid			
	Individualized Consideration	0,907	Valid			
	Intellectual Stimulation	0,879	Valid			
*Affective Org*. *Commitmet*	AOC composite	0,934	Valid	0,872	0,872	Reliable
*Job Self-efficacy*	Magnitude	0,899	Valid	0,814	0,911	Reliable
	Generality	0,905	Valid			
*Job Satisfaction*	Enjoying Work	0,908	Valid	0,774	0,897	Reliable
	Positive Work Morale	0,874	Valid			
	Discipline & Work Performance	0,856	Valid			
*Organizational Citizenship Behavior*	Alturism	0,832	Valid	0,616	0,888	Reliable
	Conscientiousness	0,832	Valid			
	Sportsmanship	0,648	Valid			
	Civic Virtue	0,817	Valid			
	Courtesy	0,779	Valid			

**Fig 3:**
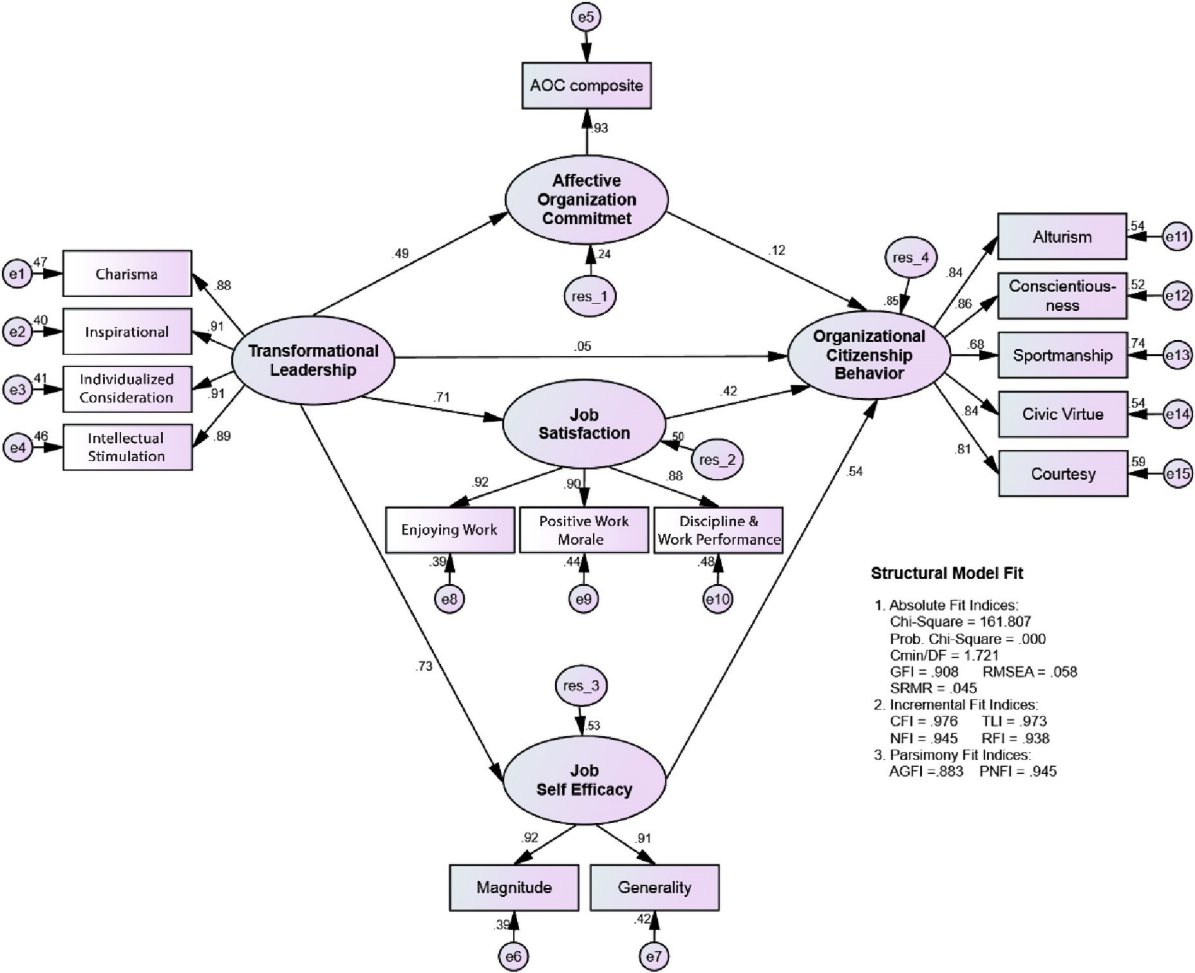
SEM estimation results with composite indicator.

The results of the calculation of the goodness of fit index values produced by the structural model are shown in Fig 3 and Table 4:

**Table 4:** Fit measure for the structural model.

*Fit Measure*		*Structural Model*	*Critical Value*	Decision
*Absolute Fit Indices*	Probability	0,000	> 0,05	*Even good fit*
	Cmin/DF	1,721	≤ 2,00	*Good fit*
	GFI	0,908	≥ 0,90	*Good fit*
	RMSEA	0,058	≤ 0,08	*Good fit*
	SRMR	0,045	≤ 0,05	*Good fit*
*Incremental Fit Indices*	CFI	0,976	≥ 0,95	*Good fit*
	TLI	0,973	≥ 0,95	*Good fit*
	NFI	0,945	≥ 0,90	*Good fit*
	RFI	0,938	≥ 0,90	*Good fit*
*Parsimony Fit Indices*	AGFI	0,883	≥ 0,90	*Marginal fit*
	PNFI	0,945	≥ 0,90	*Good fit*

Table 4 shows the results of the structural model suitability test showing that all criteria for absolute fit indices, incremental fit indices, and parsimony fit indices have met the requirements (marginal fit and good fit), the probability chi-square value is also included in the even good fit category, so the model Structural structure has been accepted, and then tested the significance of the influence between variables, both direct and indirect effects.

Table 5 shows the testing of structural relationships, hypothesis testing is said to be significant between variables using the critical ratio (CR) value and the probability value (p-value). It is said that there is a significant effect between variables if the CR value 1.96 or p-value 5% significance level, and vice versa.

**Table 5:** Summary of the direct effect hypotheses.

Hyp.	Structural Relationship			*Std*. *Estimate*	C.R.	*P value*	Inf.
H_1_	*Transformational Leadership*	→	*Organizational Citizenship Behavior*	0,047	0,620	0,536	ns
H_2_	*Transformational Leadership*	→	*Affective Organization Commitmet*	0,489	7,232	0,000	s
H_3_	*Transformational Leadership*	→	*Job Satisfaction*	0,708	12,848	0,000	s
H_4_	*Transformational Leadership*	→	*JSE*	0,729	13,320	0,000	s
H_5_	*Affective Organization Commitmet*	→	*Organizational Citizenship Behavior*	0,115	2,507	0,012	s
H_6_	*Job Satisfaction*	→	*Organizational Citizenship Behavior*	0,415	7,192	0,000	s
H_7_	*Job Self-efficacy*	→	*Organizational Citizenship Behavior*	0,538	8,686	0,000	s

Description: s (significant); ns (not significant)

Table 6 shows that the direct effect of exogenous variables on endogenous variables is significant, and the indirect effect through intervening variables is also through a significant path, then it is said to be partially mediation. On the other hand, if the direct effect of the exogenous variable on the endogenous variable is not significant, while the indirect effect of the intervening variable is through a significant path, then it is said to be fully mediation or perfect meditation.

**Table 6:** Summary of the indirect effect hypotheses.

Hyp.	Indirect Line	*Estimate*	*SE*	C.R.	*P-value*	Mediator properties
H_8_	TFL → AOC → OCB	0,052	0,023	2,322	0,022^(s)^	*Fully mediation*
H_9_	TFL → JS → OCB	0,273	0,044	6,233	0,000^(s)^	*Fully mediation*
H_10_	TFL → JSE → OCB	0,364	0,050	7,209	0,000^(s)^	*Fully mediation*

TFL: *Transformational Leadership*

AOC: *Affective Organization Commitment*

JS: *Job Satisfaction*

JSE: *Job Self Efficacy*

OCB: *Organizational Citizenship Behavior*

Description: s (significant); ts (not significant)

### 4.2 Discussion

Based on the data processing and analysis that has been carried out, in the discussion of this study, the results of the estimated parameter of the effect of TL on OCB show an insignificant effect (CR = 0.620, p-value = 0.536, SE = 0.047) which indicates the better TL. still not able to give a real impact on improving OCB. Thus, the first hypothesis is rejected. These findings are not in line with previous studies which state that there is a positive relationship between TL and OCB has been empirically supported [4, 29, 34]. These findings indicate that TL does not directly influence the extra-role behavior of prison officers such as OCB.

Then the estimation results of the TL effect on affective organization commitment showed a significant effect (CR = 7.232, p-value = 0.000, SE = 0.489). This shows that the better the TL, the stronger the affective organization commitment. Thus, the second hypothesis is accepted. This finding is in line with previous research which has revealed that TL has a direct influence on affective commitment [6, 8, 35]. The results of this study indicate that transformational leaders have a tremendous influence on the success of officers in the seven Nusakambangan prisons in building emotional attachment. In addition, this shows that transformational leaders can create meaning for their subordinates to survive in their work.

Furthermore, the results of the estimation of the TL effect on JS also showed a significant effect (CR = 12.848, p-value = 0.000, SE = 0.708). This shows that the better the TL, the higher the JS. Thus, the third hypothesis is accepted. It is known that the results of this study are in line with previous studies, which states that TL has an impact on increasing JS [21, 36, 37]. The results of this study indicate that transformational leaders will be able to directly increase the JS of the seven prison officers in Nusakambangan. Transformational leaders will make the officers of the seven prisons in Nusakambangan feel that their work needs are being met and JS will increase.

The estimation results of the TL effect on JSE also show a significant effect (CR = 13.320, p-value = 0.000, SE = 0.729) which indicates the better TL, the higher the JSE. Thus, the fourth hypothesis is accepted. It is known that the results of this study are in line with previous research, which suggested that transformational leaders are important determinants of follower self-efficacy [14, 18, 28]. The results of this study indicate that transformational leaders will be able to encourage the prison officers in Nusakambangan to rethink the demands of change, demands that may seem less threatening and officers at work, so that they will feel better able to handle these challenges. Furthermore, through the influence of TL, officers will receive support to increase their JSE.

Next, the parameter estimation results of the influence of affective organization commitment on OCB showed a significant effect (CR = 2.507, p-value = 0.012, SE = 0.115) and showed that the stronger affective organization commitment, the higher the OCB. Thus, the fifth hypothesis is accepted. Tis result is in line with previous research which stated that affective commitment determines variables that affect performance such as OCB [40–42]. The results of this study indicate that with a high level of affective commitment, officers will be more likely to be involved in OCB because they feel an emotional attachment to their organization and are willing to take extra roles at work.

The results of the estimated parameter of the effect of JS on OCB also show a significant effect (CR = 7.192, p-value = 0.000, SE = 0.415). This indicates that the higher the JS, the higher the OCB. Thus, the sixth hypothesis is accepted. This result is in line with previous research which states that employees who enjoy higher levels of JS will be able to show higher levels of OCB [22, 44]. The results of this study indicate a pleasant emotional state resulting from the assessment of the work of the seven prison officers in Nusakambangan as an achievement or facilitation of achieving the value of their work which in turn provides a willingness to OCB from the officers.

The estimation results of the parameter of the effect of JSE on OCB also show a significant effect (CR = 8686, p-value = 0.000, SE = 0.538) this indicates that the higher JSE, the higher OCB will be. Thus, the seventh hypothesis is accepted. It is known that the results of this study are in line with previous researchs which have confirmed that self-efficacy leads to OCB [26, 47, 48]. The results of this study indicate that officers in the seven prisons in Nusakambangan who have high self-efficacy tend to be more confident because they believe he can complete his work, then they will dare to take action and maximize their abilities, including leading to a willingness to do work outside of their obligations such as OCB.

The results of the TFL→AOC→OCB indirect path significance test showed a significant effect (CR = 2.322, p-value = 0.022, SE = 0.023) so, the eighth hypothesis is accepted. The nature of the mediator is known to be fully mediation, meaning that increasing OCB in officers cannot rely on TL directly, but it is necessary to increase the affective organization commitment of officers to transmit this goal. The results of this study indicate that the role of TL will have an effect on the OCB of officers through the affective commitment they feel towards the organization. Thus, it is stated that when employees have high-quality social exchange relationships from the influence of TL on the organization, the affective commitment of the seven prison officers in Nusakambangan increases, and they are more likely to be involved in extra-role behavior or OCB.

The results of the TFL→JS→OCB indirect path significance test showed a significant effect (CR = 6.233, p-value = 0.000, SE = 0.044). Thus, the ninth hypothesis of this study is accepted. The nature of the mediator is known to be fully mediation, meaning that increasing OCB in officers does not have an impact on transformational leaders, but must also increase employee JS first. The results of this study indicate that JS can transmit TL which then affects the willingness of officers regarding OCB. Thus, these results show that transformational and OCB leaders from the seven prison officers in Nusakambangan will be able to connect with the JS they feel.

The results of the TFL→JSE→OCB indirect path significance test also showed a significant effect with the value (CR = 7.209, p-value = 0.000, SE = 0.050). Thus, the tenth hypothesis is accepted. The nature of the mediator is also known to be fully mediation, meaning that TL will not be able to make officers willing to show OCB without JSE as a liaison, this happens because the path can only be indirect. The results of this study indicate that self-efficacy from the prison officers in Nusakambangan can transmit the effects of TL in creating positive behavior voluntarily in performing extra roles such as OCB.

## 5. Conclusion

Based on the results, it can be concluded that there is insignificant influence of TL on OCB, significant effect of TL on AOC, significant effect of TL on JS and JSE, significant effect of AOC, JS and JSE on OCB, and significant mediation role of JS and JSE to link TL to OCB. It is known that OCB is very important for all organizations because it is expected to encourage progress and achieve company goals. From the conclusion of this study, it shows that the officers involved are able to be motivated to act on OCB. This can happen because of the influence of TL that makes officers feel AOC, JS, and JSE which leads to an increase in OCB in creating good and appropriate job quality. Thus, the officers involved will be considered beneficial to the organization that cannot be added on the basis of formal role obligations and with recommendations through extra roles in doing work.

## 6. Implications

### 6.1 Theoretical implications

This research highlights the positive behavior of human resources in creating discretionary behavior that goes beyond formal job descriptions to increase organizational effectiveness and add meaning to the daily work of working employees. The model from this study complements the existing literature by highlighting the importance of transformational leadership to lead to positive results that will be provided by these employees. Furthermore, this research is expanded by adding roles through affective organizational commitment, job satisfaction, and job self-efficacy which act as mediation. Former research found that transformational leadership strengthened organizational citizenship behavior in working individuals through the emotional attachment to the organization. This research proves that correctional officers who have affective commitment will voluntarily demonstrate OCB [35]. Thus, the results of this study expand the literature in the context of correctional officers. In addition, transformational leadership is able to influence officers who are satisfied with their work leading to OCB. In line with the statement, individuals who work under the influence of transformational leadership will be more satisfied and committed to their organizations and show positive behavior for the organization [49].

The findings reinforced by the statement that correctional officers with higher levels of job satisfaction will display more pro-social behavior, such as organizational citizenship behavior [22]. The results of this study additionally enrich the current literature about the effect of transformational leadership on organizational citizenship behavior. Previous study has demonstrated that self-efficacy is a transformational leadership mechanism that can improve attitude change to attain the desired results [14]. The results of this study have showed that job self-efficacy can influence correctional officers to better comprehend the effect of leaders with a transformational style to willingly take on extra roles such as organizational citizenship behavior.Besides that, this research contributes to a better understanding of extra-role behavior based on social exchange theory which shows that individuals will have a tendency to repay dyadic partners with behaviors that are beneficial to them, and in an organizational context. This behavior can be shown through organizational citizenship behavior, in which employees lead to activities that are not part of their responsibilities but help the organization to achieve its goals. Therefore, regardless of how they view their immediate supervisor’s transformational leadership behavior, employees may be more likely to engage in affective organizational commitment, job satisfaction, and job self-efficacy which will lead to discretionary extra-role behavior. In addition, this study also highlights the different mechanisms in the research design using longitudinal data spread over three periods of data distribution at different times with a difference of two weeks, and multiple studies intended for robustness of planning, process and research results.

### 6.2 Empirical implications

This study highlights the effect of TL on behaviors that have an important impact on extra-role behaviors required in the work context. Contributions to the literature are obtained from research results which show that the three mediations namely JSE, AOC and JS in parallel fully mediate the influence of TL on OCB. The findings of this study indicate that the role of an inspirational leader will make an employee happy to provide assistance to his colleagues when the employee enjoys his work, has high capabilities in various jobs, and feels part of the organization where he works. Thus, this research shows the importance of leadership that is able to provide intellectual stimulus and awareness to subordinates on the importance of having confidence in solving various problems. In addition, leadership that gives attention will also be able to make employees feel recognized and feel that problems within the organization are the problem. Furthermore, this will make employees more able to adapt to changes that occur in the scope of their work by showing a positive contribution in helping problem solving.

In addition, this study contributes to a better understanding of the implications of extra-role behavior based on theoretical support for social exchange theory which shows that individuals will have a tendency to reciprocate dyadic interactions with behaviors that are beneficial to them, and in an organizational context. This behavior can be demonstrated through OCB, where employees lead to activities that are not part of their responsibilities but help the organization to achieve its goals. Therefore, regardless of how they perceive the TL behavior of their immediate supervisor, employees may be more involved in AOC, JS, and JSE which will lead to discretionary extra-role behavior. In addition, this study also highlights different mechanisms in research design using longitudinal data that is distributed in three periods of data distribution at different times with a time difference of 2 weeks, and multiple studies which are intended for robustness of research planning, processes, and results. Thus, this study provides additional knowledge and understanding of the consequences of TL on important attitudes and behaviors in the survival of organizations that have limited human resources and are faced with dynamic situations.

### 6.3 Practical implications

Based on the results of the research that has been described regarding the effect of TL through AOC, JS, and JSE on OCB on the officers of the Nusakambangan Prison Complex, there are several suggestions, namely that the organization is expected to encourage a leadership style that provides stimulation, attention, and inspiration. can create positive behavior related to OCB when officers have an attitude within themselves that encourages confidence in their ability to take action to achieve certain goals. In addition, leaders who provide stimulation, attention, and inspiration will make officers have satisfaction with their work and show great commitment to their work which in turn will have extra role behaviors that go beyond formal job descriptions in increasing organizational effectiveness which also adds meaning to daily work. -days for organizational contributions. In addition, this study also provides practical advice for managers to apply a TL style because of its positive impact, influencing the attitudes of workplace officers that encourage officers to have extra-role behavior at work. Thus, the results of this study provide a comprehensive understanding of the effect of TL on OCB in the context of public organizations, especially prison institutions.

### 6.4 Limitation of the study

There are several limitations that may be of concern to further research to complement this research. First, the sample size is limited according to the number of available research subjects. Future research may collect data from other cities and other public sector organizations. Second, future research can use the same variables but to test the characteristics of different objects, either from sample sizes, organizational forms or different contexts to broaden and deepen knowledge in the field of human resource management. Third, modifications can be made, for example by reducing, adding or changing the variables to obtain a broader research theory. This study also does not include a moderating role that could affect this relationship. Therefore, future research needs to involve the role of moderating variables. In addition, this study measures variables solely from the officer’s perspective. Therefore, future researchers can do similar studies using data from diverse perspectives, such as those of leaders and peers.
